# Comparison of bone formation mediated by bone morphogenetic protein delivered by nanoclay gels with clinical techniques (autograft and InductOs^®^) in an ovine bone model

**DOI:** 10.1177/20417314221113746

**Published:** 2022-09-16

**Authors:** Cameron Black, David Gibbs, Josephine McEwan, Janos Kanczler, Marta Peña Fernández, Gianluca Tozzi, Jonathan Dawson, Richard Oreffo

**Affiliations:** 1 Bone & Joint Research Group, Centre for Human Development, Stem Cells and Regeneration, Human Development & Health, Institute of Developmental Sciences, University of Southampton, Southampton, UK; 2 Institute of Mechanical, Process and Engineering, School of Engineering and Physical Sciences, Heriot Watt University, Edinburgh, UK; 3 Zeiss Global Centre, School of Mechanical and Design Engineering, University of Portsmouth, Portsmouth, UK; 4 College of Biomedical Engineering, China Medical University, Taichung, Taiwan

**Keywords:** Nanoclay, InductOs^®^, bone morphogenic protein-2 (BMP-2), ovine, condyle defect

## Abstract

Development of a growth factor delivery vehicle providing appropriate temporal-spatial release together with an appropriate preclinical large animal model to evaluate bone formation is critical in the development of delivery strategies for bone tissue regeneration. Smectite nanoclays such as LAPONITE™ possess unique thixotropic and protein retention properties offering promise for use in growth factor delivery in bone repair and regeneration. This study has examined bone formation mediated by a clinically approved growth factor delivery system (InductOs®) in combination with Laponite gel in an aged female ovine femoral condyle defect preclinical model (10 weeks). Two different designs, one containing a low volume of Laponite gel (LLG) in combination with the InductOs® absorbable collagen sponge (ACS), the other in which Laponite gel formed the implant (HLG), were compared against InductOs® alone and an autograft positive control. Thus, five groups: (i) empty defect, (ii) autograft, (iii) BMP2 + ACS, (iv) BMP2 + ACS + LLG and (v) BMP2 + HLG + ACS were examined in 9 mm × 12 mm defects performed bilaterally in the medial femoral condyles of 24 aged (>5 years) sheep. Bone formation within the defect was assessed using micro-computed tomography (micro-CT), digital volume correlation (DVC) for biomechanical characterisation as well as histology. The autograft and InductOs® mediated enhanced bone formation (*p* < 0001) compared to blank controls, while no significant differences were observed between the Laponite/Collagen/BMP delivery vehicles. However, the current study illustrated the excellent biocompatibility of Laponite and its ability to deliver localised active BMP-2, with the opportunity for improved efficacy with further optimisation. Interestingly, DVC-computed strain distributions indicated that the regenerated bone structure is mechanically adapted to bear external loads from the early remodelling stages of the bone reparation cascade. The current studies of selected nanoclay delivery platforms for BMP, assessed in a clinically relevant large animal model auger well for the development of bone fracture therapeutics for an ageing population.

## Introduction

Bone morphogenetic proteins (BMP) are powerful stimulators of osteogenesis and play a pivotal role in the regulation of bone formation in humans and other species. With the exception of BMP-1, these molecules are part of the Transforming Growth Factor-*beta* superfamily with BMP-2, 4, 6 and 7 considered osteoinductive. The osteoinductive capacity resides in the critical role of the BMPs in skeletal stem cell recruitment, commitment, and osteoblast differentiation enhancing ossification.^1^ Over 20 subtypes of BMP have been defined^1^ of which 2 have been licenced for human use for over a decade; BMP-2 for stimulation of spinal arthrodesis (Infuse, InductOs^®^) and fracture union (InductOs^®^), and BMP-7 (OP-1 Stryker) to enhance fracture union.^2^

The efficacy of BMP to mediate tibial fracture union and spinal arthrodesis has been shown in clinical trials.^2^ Initially, this technology received significant attention, as BMP therapy heralded an opportunity to avoid the use of autologous graft, usually required in these clinical scenarios. Autograft, typically harvested from the iliac crest has significant limitations, namely autograft harvest involves an additional surgical procedure, volume available is limited and harvesting frequently results in post-operative pain and in some cases infection.^3,4^

The current approach for clinical delivery of BMP typically incorporates dissolving lyophilized BMP in saline, which is subsequently applied to a bovine derived collagen sponge and applied to the target site (InductOs^®^) during open surgery.^5^ A dose of 6–12 mg of BMP is typically used, which is several orders of magnitude greater than physiological quantities (200 ng/ml BMP) required to elicit a cellular response.^6^ The quantities of BMP required, in part due to inefficient delivery mechanisms, have generated a largely uneconomic clinical product^2^ and, crucially, significant side effects associated with BMP use at such pharmacological concentrations. Critical side effects associated with poor BMP localization include: swelling, which have on occasion resulted in airway compromise when used in cervical spinal fusion^7^ and heterotopic ossification resulting in compression of nerves^8^ and osteolysis.^9^ In the mainstay, this promising therapy has recently fallen out of favour as a consequence of the large doses required clinically together with suboptimal delivery mechanisms resulting in an unacceptable risk/benefit profile.

Certain clays, such as Laponite form colloidal gels that possess unique protein retention properties, suggesting their utility for safer and more effective delivery of growth factors such as BMP.^6,10^ In addition to the useful thixotropic nature of Laponite which means it can be delivered via a needle to the target site, the ability to bind and sustain localised concentrations of BMP2 within the defect environment suggest a key advantage that may address the limitations associated with current collagen sponge based carriers.

Clays have been widely used in the pharmaceutical industry both as excipients and active substances and generally exhibit a good biocompatibility profile.^11^ Laponite is a synthetic product based on the naturally occurring smectite clay, hectorite and is layered magnesium lithium silicate in which tetrahedral silica layers sandwich a central octahedral sheet.^11^ Upon hydration in water, Laponite disperses to form 1 nm thick platelets of approximately 25 nm diameter. The dual surface charges of these platelets interact in water to generate reversible (thixotropic) gel states. Laponite discs display a broad spectrum affinity for protein binding via a variety of mechanisms including electrostatic interactions cation exchange, hydrophobic and interlamellar mechanisms.^12^ These properties suggest promising utility for therapeutic protein delivery through extended release or, indeed, site-specific localisation. Critically, no toxicity has been observed by Laponite in a number of *in vitro* and *in vivo* studies.^6,12,13^ Our group has previously demonstrated that nanoclays are effective at binding and delivering growth factors to sustain localised concentrations *in vivo* and have applied this approach to initiate the formation of new blood vessels at an injury site through localisation of vascular endothelial growth factor^12,14^ and to induce bone at the lowest dose of bone morphogenetic protein (BMP)-2 published in the literature to date.^6,15^

While murine models of ectopic bone formation provide an efficient, low cost rapid method of *in vivo* assessment of growth factor delivery vehicles to enhance bone formation^6^; the lower surface area to volume ratio of bone formation in murine models limits the application of this data to clinical practice. Molecular signalling, nutrient availability and cellular infiltration observed with murine surface area to volume ratio may be critically different when scaled to clinically relevant dimensions. Furthermore, bone healing in rodents is rapid, and almost universal, which is distinct from humans. The ovine femoral condyle defect model provides a preclinical model for testing tissue engineering strategies at a clinically relevant scale. Recent studies have employed a model homologous to that published by Ding et al.^16^ and advised from detailed protocols by Nuss et al.^17^ In an advancement of the published protocols, a model was developed using only an aged female population, more representative of the clinical demographic in humans at risk of fragility fractures. All animals used were female, and at least 5 years of age, in contrast to the typical use of animals 24 months of age. Ovine condyle models have previously been used in examination of novel biomaterials, biomedical devices and orthopaedic implants.^18–20^

Bone formation at a fracture or arthrodesis site is a key factor which is imperative in the healing process. As such, bone formation within the ovine condyle assessed using micro-Computed Tomography (micro-CT) was selected as the primary endpoint and this is consistent with previous work.^21^ While bone volume is of relevance, bone volume fails to take into account bone microarchitecture which changes as healing advances from isotropic to anisotropic, nor does bone volume directly relate to biomechanical strength. From a clinical perspective, biomechanical strength at the fracture or arthrodesis site is critical. Biomechanical strength sufficient to withstand normal physiological loading enables full mobilisation without risk of fracture/arthrodesis displacement or fixation failure. Additionally, tissue differentiation during fracture healing is known to be mechano-regulated, both by the shear strain of the solid phase (i.e. bone) and the interstitial fluid velocity driven by the pore deformation.^22^ Therefore, in a subgroup we used digital volume correlation (DVC) to facilitate advanced characterisation of microarchitecture and biomechanics of regenerated bone within the defect via determination of 3D full-field strain distribution.^23^ The combination of micro-CT, histology and DVC was utilised to enable a comprehensive analysis of bone volume, micro architecture and mechanical function formed within the defect in response to control and test formulations.

This study aimed to compare the ability of BMP delivered with a synthetic nanoclay, Laponite, to produce bone within a defect site in comparison with clinical techniques: autograft, InductOs® and blank control defects.

## Materials and methods

Laponite was obtained from BYK Widnes, UK. Rh-BMP-2 & type 1 collagen sponge -InductOs®, Medtronic. All other regents were obtained from Sigma Aldrich, UK, unless stated. Laponite preparation: Clay gels were prepared as described previously.^6^ Briefly, Laponite XLG (BYK, Widnes, UK) was dispersed in distilled water (dH_2_O) at a concentration of 2.5 wt. % (25 mg/ml) under rapid agitation. The preparations were subsequently sterilized by autoclave and evaporated water replaced with sterile dH_2_O.

### Sample size calculation

This study comprised five experimental groups: (i) empty defect - blank control, (ii) autograft, (iii) InductOs®-BMP-2 absorbable collagen sponge (ACS), (iv) BMP2 + ACS + LLG, (LLG – Low volume Laponite) and (v) BMP2 + HLG + ACS (HLG – High Volume Laponite). The primary outcomes for the studies was the bone volume/tissue volume ratio within the defect (BV/TV, at 10 weeks). Based on previous work^21^ of bone formation in tibial and femoral defects of 5 mm diameter and 15 mm depth, which saw mean effect sizes of 15 ± 9% (BV/TV ± SD) between highest and lowest groups, we calculated that the same effect would be detectable with 80% power at a 5% type error rate with *n* = 6 samples per group.

### Scaffold design and preparation

Two different preparations combining 2.5 wt. % Laponite gel with InductOs® absorbable collagen sponge (ACS), were compared against controls differing in the ratio of Laponite gel to ACS in the bulk scaffold. LLG containing a lower volume of Laponite gel consisted of a layered conformation of ACS + BMP2 sandwiching two volumes of Laponite gel while HLG, which contained a higher volume of Laponite gel was a combination of BMP2 + Laponite injected directly in to the bone defect before being ‘capped’ with ACS. These experimental treatments were compared against an ACS + BMP2 treatment without Laponite, an autograft positive control and an empty defect negative control.

**Figure 1.**
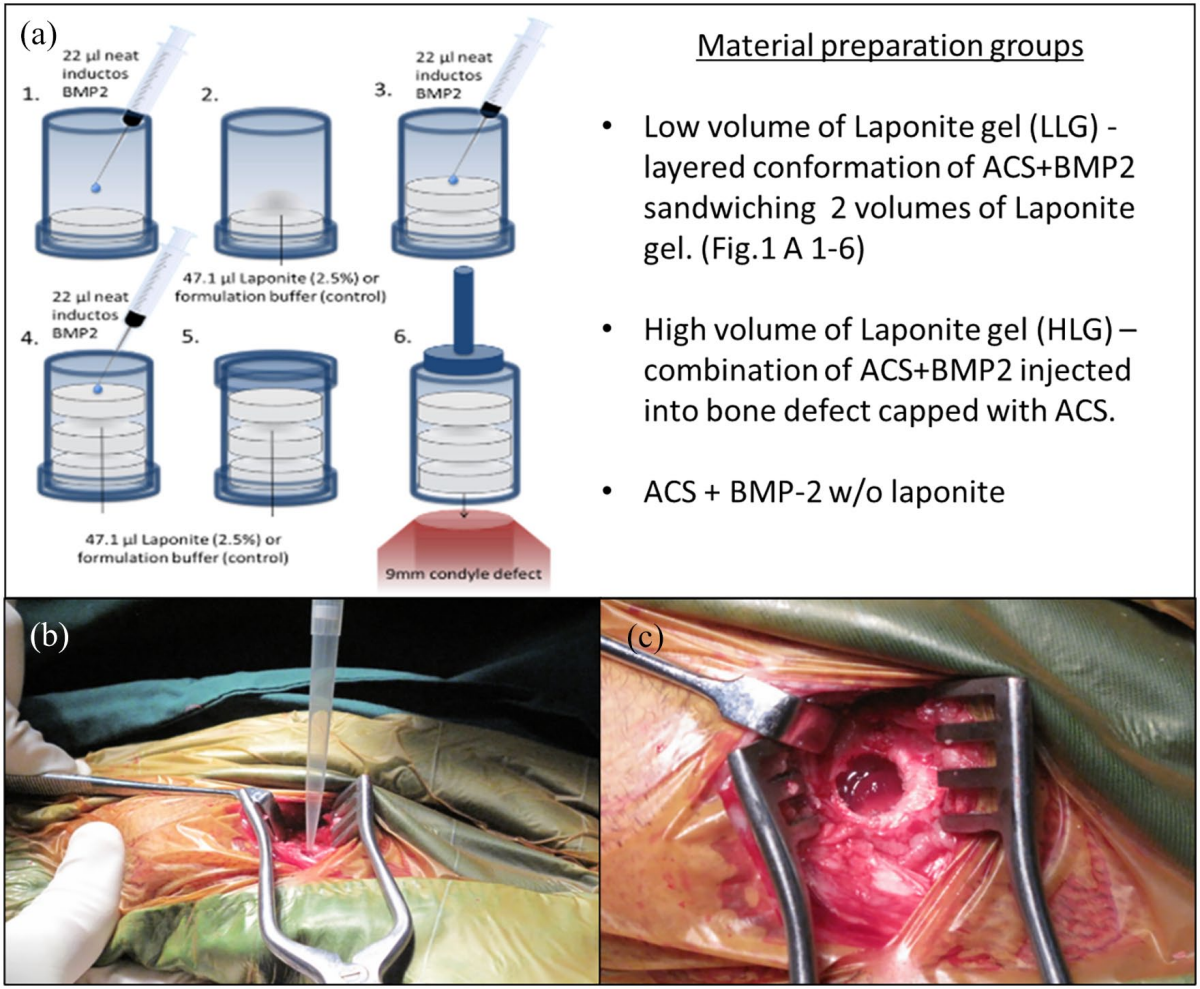
Image (a) shows the scaffold construction for treatment groups BMP2 + ACS + LLG, BMP2 + HLG + ACS and BMP2 + ACS detailing the Laponite (LLG or HLG) layered conformation with ACS+BMP2 sandwiching two volumes of Laponite Figure 1(a) (1) InductOs® BMP2 was added onto the ACS according to manufacturer’s instructions while contained within a supportive 8 mm diameter tube with a removable cap on the base. (2) 2.5% Laponite was layered onto the first layer of BMP2 loaded ACS. (3) 2.5% Laponite was sandwiched between a second ACS to which InductOs® BMP2 was added as above. (4) Steps 2–3 were repeated and (5) enclosed within the tube for transport to the surgical field. (6) After removing both caps implantation was achieved using a syringe plunger to transfer the assembled scaffold into the defect. Image (b) shows the treatment Laponite with BMP-2 being directly pipetted into the condyle defect. Image (c) shows the Laponite with BMP-2 treatment *in situ* in the bone defect. Prior to suturing the gel was capped with a collagen sponge disk.

To prepare BMP2 + ACS + LLG three stacked disks of ACS + BMP2. Three 4 mm × 8 mm ACS discs were prepared from the supplied collagen sheet (InductOs^®^) using an 8 mm diameter biopsy punch (Kruuse UK LTD). ACS-Laponite constructs were assembled by placing a disc of ACS in a mould (9 mm × 12 mm) and subsequently adding 22 µl of BMP-2 (1.5 mg/ml BMP-2), followed by 47.1 µl of 2.5% Laponite. A second layer of ACS, BMP-2 and Laponite was repeated identical to the first. A final third ACS was added with BMP-2 neat applied to the top layer (without Laponite). Thus, a total of 66 µl of BMP-2 (100 µg) was applied per construct, and with each defect containing 100 μg of InductOs® BMP-2 (Figure 1(a)). The InductOs^®^ only control was prepared in an identical manner but formulation buffer was used in place of Laponite gel. The alternative preparation, BMP2 + HLG + ACS, applied a defect filling volume (603 µl) of Laponite gel + 100 µg BMP2 directly into the defect (Figure 1(b)) before capping with a single ACS disk. Here, in contrast to BMP2 + ACS + LLG, BMP2 was combined with Laponite gel prior to addition of ACS. After preparation, scaffold constructs or components were stored sterile on ice and transferred to surgery.

### Ovine condyle defect model

Bone Defects (9 mm diameter × 12 mm) were performed bilaterally in the medial femoral condyles of 24 aged (>5 years) Welsh Upland Ewes (60–75 kg) under ethical approval, Home Office licence PPL30/2880. A 3 cm incision over the medial femoral epicondyle was performed and the bone defect was produced using a Osteomed OsteoPower® orthopaedic drill with a trephine drill bit (Primadent®, UK), 9 mm external diameter and 12 mm in depth. Bone cores were removed using a custom made tool developed at the University of Southampton. Autograft was prepared intraoperatively under aseptic conditions from medial femoral condyles. After the surgical creation of the defect, two bone cores (9 mm × 12 mm) were morselised using hand bone ronguers, resulting in a working graft, which was then applied to the defect. All constructs were implanted into bone defects within 30 min of assembly. The defect area was inspected for the presence of a stable defect floor, bleeding and trabecular density. At this stage, the defect is completed and either remains empty (blank control), filled with intervention, or filled with autograft material (positive control). The groups of sheep condyle defects comprised of; Empty defect (*n* = 7); Autograft implants (*n* = 10); BMP-2 + ACS (*n* = 11); BMP-2 + ACS + LLG (*n* = 9) and BMP-2 + HLG + ACS (*n* = 9). The wound was closed in layers using vicryl sutures. Sheep were recovered from anaesthesia, and placed on a customised recovery trolley with restricted movement (approximately 15–20 min). Once sheep were fully ambulatory, they were returned to their pens, freely moving and fully loadbearing. Sheep underwent euthanasia and sample sites retrieved 10 weeks post implantation (Figure 1). The femoral condyle defect samples were then fixed in 4% paraformaldehyde (PFA) and then imaged using micro-CT.

### Micro-CT image acquisition, reconstruction and analysis

Samples were removed from the specimen containers and wrapped in cling film to prevent desiccation. Wrapped samples were placed in the Skyscan 1176 micro-CT scanner (Bruker, USA) and imaged at 90 kV, 278 µA, with a 0.1 mm Cu filtre and a voxel size of 18 μm. Image segmentation for bone morphology was carried out using mean Otsu threshold.^24^

#### Volumes of interest (VOI)

Images were rotated using Data Viewer Software (Skyscan, Bruker) to obtain a view centred on the defect. Due to the spherical nature of the condyle the most superficial part of the defect is often not fully enclosed. The top of the enclosed ROI for the defect was manually selected as the first axial slice with a complete circle of bone. The bottom axial slice ROI was taken to be 25 slices (approx. 0.5 mm) superficial to the first slice at which the trabecular pattern was unbroken, when moving from the defect to the surrounding bone. This cylindrical volume enclosed within these two ROI was named as enclosed defect VOI. A mini cylinder within the enclosed defect was produced using the software to select a 4 mm × 4 mm cylinder centrally from the first slice fully enclosed by bone. Raw data were reconstructed using NRecon software v.1.6.10.4, correcting for beam hardening (30%), ring artefacts, and misalignment. CTAn software v.1.16 was used to visualize and analyze the reconstructed images for bone volume (BV)/tissue volume (TV) ratio.

### Histology

After the completion of micro-CT scanning, reconstruction and analysis, the femoral condyle samples (*n* = 6–9) were trimmed in size using a low speed precision Isomet^TM^ sectioning saw (Buehler, UK). The sample was then cut directly down the central region of the defect site of the femoral condyle and imaged using a Canon 10 digital camera. One-half of the sample was then decalcified using 10% Ethylenediaminetetraacetic acid (EDTA)/ TRIS/HCL pH7.4. The solutions were changed every 72 h and decalcification was monitored using an Faxitron MX-20 X-ray machine (Qados, UK).

Following micro-CT analysis and decalcification, the femoral condyle defect samples were dehydrated through a series of ethanol washes (50%, 90%, and 100% in dH2O) and incubated in Histo-Clear (National Diagnostics). Following ×2 incubations in paraﬃn wax for 1 h at 60°C, samples were embedded in wax blocks using an automated Shandon Citadel 2000. Consecutive 7 μm thick sections were cut throughout the depth of the central region of the femoral condyle defect placed on histology glass slides and dried at 37°C for 4 h. Mounted sections were rehydrated through Histo-Clear, graded ethanol’s (50%, 90%, and 100% in dH_2_O) and dH_2_O ready for staining. Sections were stained for the nuclear counterstain Weigert’s haematoxylin followed by staining with 0.5% Alcian blue 8GX for proteoglycan-rich cartilage matrix and 1% Sirius red F3B for collagenous matrix. Alcian blue/Sirius Red stained tissue sections were also imaged for birefringence using a Zeiss axiovert 100 microscope.

Additionally, separate slide sections were stained for Goldner’s Trichrome to detect bone and osteoid according to standard protocols. Sections were then dehydrated and mounted with DPX before imaging with an Olympus BX-51/22 DotSlide digital virtual microscope using OlyVIA 2.1 software (Olympus Soft Imaging Solutions, GmBH).

### *In situ* mechanical testing and digital volume correlation (DVC)

Regions of the trimmed femoral condyle defect samples not used for histological investigation were used to assess the biomechanical behaviour of the regenerated bone. These samples correspond to a subset of the samples analysed in,^23^ which were here re-evaluated. In addition, a blank control sample was included for mechanical characterisation. Prior to mechanical testing, cylindrical samples (5 mm diameter × 11 mm length) were cored from the defect regions under constant water irrigation. In total, four cylindrical specimens extracted from the newly formed bone region in the defect area were analysed: blank control, autograft, BMP2 + ACS and BMP2 + ACS + LLG. These were chosen as representative bone specimens with BV/TV ranging from 22.4% to 52.2% and trabecular thickness ranging from 106.8 μm to 232.7 μm.^23^ BMP2 + ACS + HLG bone specimens could not be included due to the experimental constraints for extracting a cylindrical core containing newly formed bone from the trimmed femoral condyle defect samples.

The ends of the bone cores were trimmed plane and parallel, cleaned, covered with epoxy resin, and press-fit within cylindrical, brass endcaps that were aligned using a custom-made jig. Approximately, 2 mm of the core was embedded into each endcap and 6 mm of bone was left exposed between the endcaps. Step-wise uniaxial compression testing of the cylindrical samples in combination with time-lapsed high-resolution X-ray computed tomography (XCT) (Zeiss Versa 510, Pleasanton, CA, USA) was performed in the apparent elastic regime (CT500, Deben Ltd, UK) as reported in.^23^ Briefly, a small preload (~2 N) was first applied to ensure good end contact prior to testing and each specimen was subjected to three compression steps (i.e. 1%, 2%, and 3% of apparent compression) with XCT datasets acquired under load at each step. The X-ray source voltage and current were set to 60 keV and 84 µA, respectively. A 0.4X objective lens was used to provide an isotropic voxel size of 5 µm and 1800 projections were taken over 360° with an exposure time of 10 s.

The 3D images were rigidly registered using the unloaded images as a reference and denoised by applying a non-local means filter.^25^ The filtered XCT images were masked in order to evaluate the strain distribution only in the mineralised tissue and segmentation was performed by applying a global thresholding based on Otsu’s method.^24^

DVC software (DaVis 8.4, LaVision, Goettingen, Germany) was used to compute the full-field strain distribution for the four specimens in their apparent elastic regime. DaVis is a cross-correlation method operating on the intensity values (grey-level) of 3D images. Details of the operating principles have been reported elsewhere.^26,27^ The present DVC computation relied on a final sub-volume of 40 voxels (200 µm), reached after successive (predictor) passes using sub-volumes of 72, 64, 56, 48 and 40 voxels, with a 0% overlap. The DVC parameters used in this study relied on previous methodological work^23,27,28^ to determine strain uncertainties that for the employed scheme were constantly lower than 400µε. In order to evaluate the 3D strain distribution in the selected specimens over time in relation with the applied compressive load and bone regeneration, the maximum shear strain (γ_max_) was computed.

#### Statistical analysis

Statistical analysis was performed using Graphpad Prism 9.0. ANOVA with Tukey post hoc testing was performed on the primary endpoint of bone volume per defect volume.

## Results

### Final numbers for analysis and exclusions

**Figure 2.**
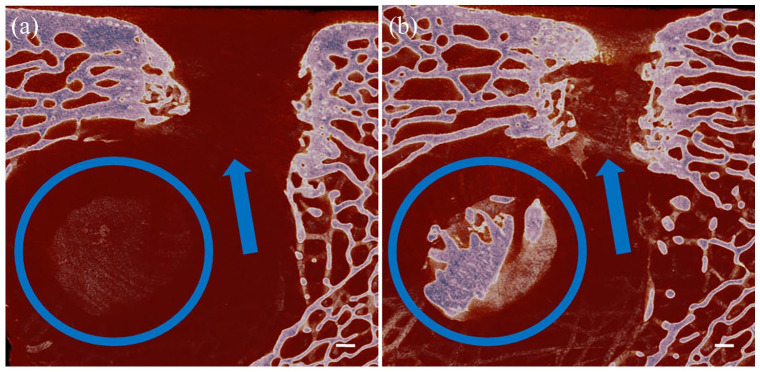
Images (a and b) are sagittal sections through the condyle defect. Both images show a condyle defect has been made that is continuous with an underlying bone void, therefore, there is no floor to the defect (indicated by the blue arrows). Scale bar = 1 mm.

A total of 24 aged Ewes underwent surgery as detailed in the methods resulting in a total of *n* = 48 defects (Table 1). A total of *n* = 12 were excluded from analysis for the following reasons: two sheep required euthanasia due to lameness (*n* = 4), in six defects the entire cortex was breached allowing displacement of test material into the medulla or a large bone void was seen immediately adjacent to the defect (*n* = 6) (Figure 2); in a further two defects the test site was sectioned during retrieval (*n* = 2).

**Table 1.** Sheep defect number per group for the study.

	Empty Defect	Autograft	BMP-2 + ACS	BMP-2 + ACS + LLG	BMP-2 + HLG + ACS	Total
Study start	*n* = 7	*n* = 10	*n* = 11	*n* = 11	*n* = 9	*n* = 48
Survived	*n* = 7	*n* = 9	*n* = 10	*n* = 9	*n* = 9	*n* = 44
Voids/perforation	*n* = 2	*n* = 1	*n* = 2	*n* = 1	-	*n* = 6
Other exclusions	*n* = 1	-	-	*n* = 1	-	*n* = 2
Analusis	*n* = 4	*n* = 8	*n* = 8	*n* = 7	*n* = 9	*n* = 36

### Bone formation

**Figure 3.**
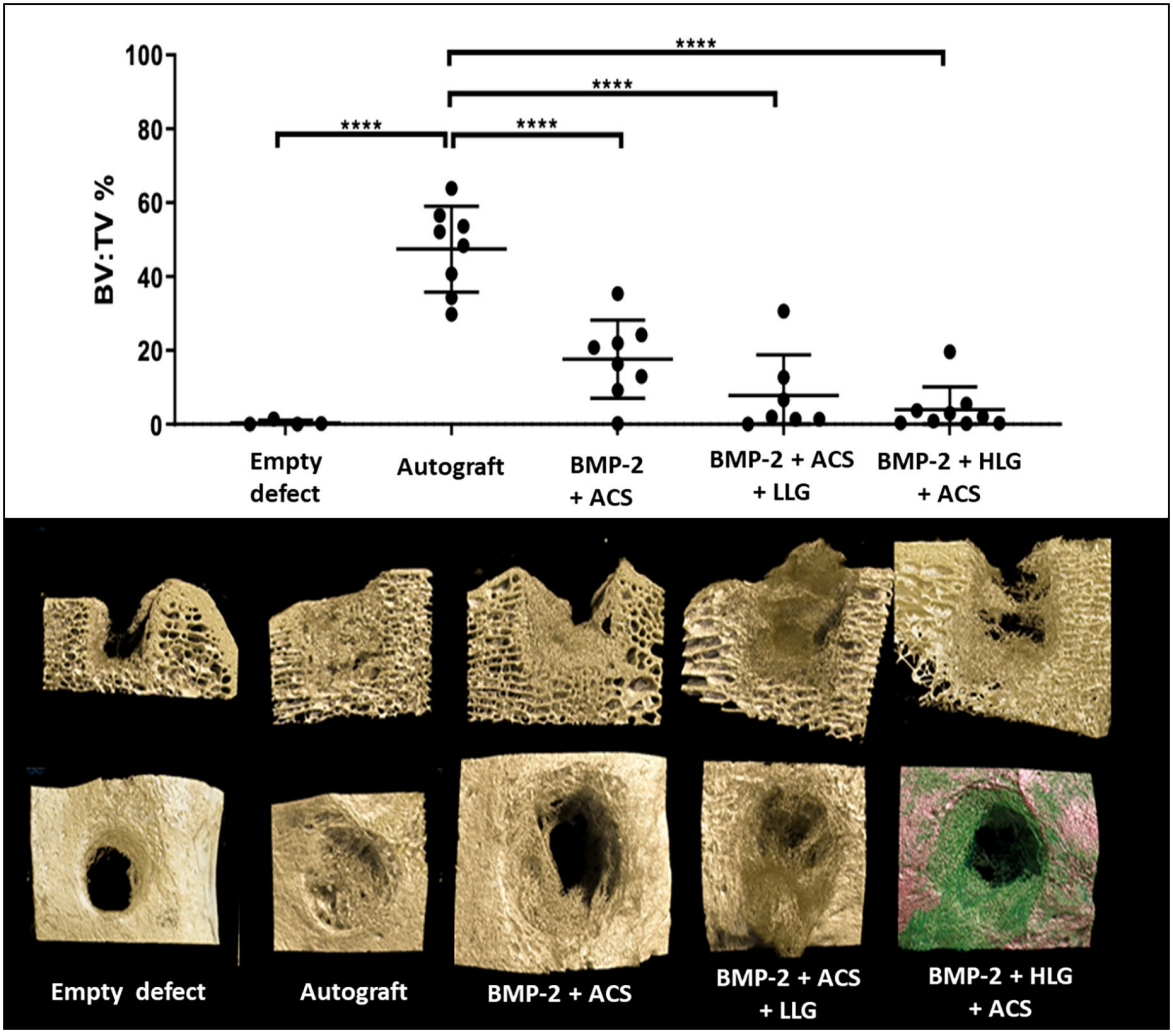
Graph demonstrating bone volume per tissue volume in the assigned mini-cylinder region of the defect, *p < 0.05 and ****p < 0.0001. Representative 3D micro-CT reconstructions in sagittal and transverse sections are shown below for each corresponding group.

Bone formation within the defect was greatest with autograft compared to blank control defects. There was no significant difference in BV/TV formed within the defect observed with BMP2 + ACS, BMP2 + ACS + LLG or BMP2 + HLG + ACS when compared to the empty defect control. Autograft resulted in significantly greater bone formation compared to: BMP2 + ACS, BMP2 + ACS + LLG or BMP2 + HLG + ACS (Figure 3).

### Histological analysis

**Figure 4.**
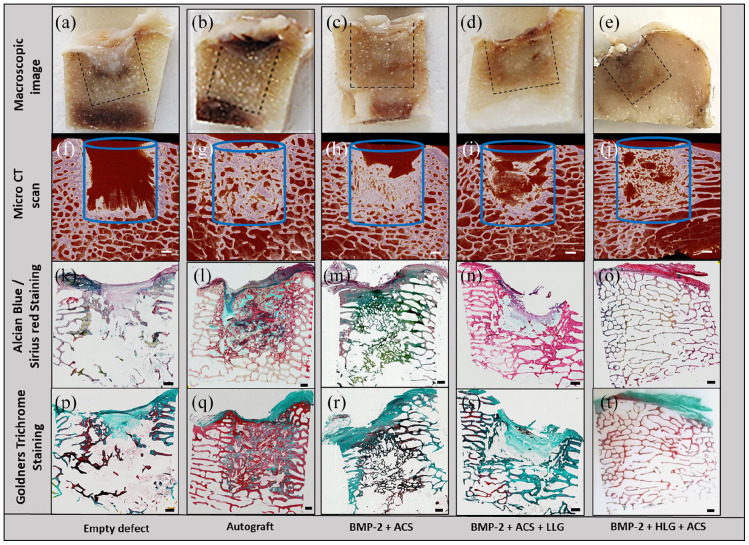
Micro-CT, macroscopic and microscopic sagittal sections of the femoral condyle defect groups. Macroscopic images of the central regions of the condyle defect from each group (a–e). Micro-CT scan sections of the defect groups (f–j). Alcian blue/Sirius Red (k–o) and Goldners trichrome (p–t) staining of condyle defect sections. Scale bar = 1 mm.

The macroscopic image of the empty defect control demonstrated a fibrous tissue in the condyle defect region (Figure 4(a)) which was negative for dense bone tissue in the corresponding micro-CT scan (Figure 4(f)). Sagittal sections of the femoral defect samples demonstrated dense compacted trabecular bone in the autograft defect samples (Figure 4(b)), which correlated with the micro-CT imaging (Figure 4(g)). The BMP2 + ACS implant group demonstrated a thin trabecular bone network (Figure 4(c) and (h)) with a fibrous cap region at the top of the defect. The BMP2 + ACS + LLG (Figure 4(d) and (i)) and BMP2 + HLG + ACS (Figure 4(e) and (j)) demonstrated similar trabecular networks but in the BMP2 + ACS + LLG, the bone was developing from the surrounding bone and growing into the implant region. A notable feature of this group, in contrast to others, was the presence of large regions of void intersected with bridging bone (Supplemental Figure 1). The BMP2 + HLG + ACS group developed bone trabeculae more uniformly across the cross-section of the defect, but these appeared to be long and attenuated. Alcian blue/Sirius red staining of the defect samples dense proteoglycan matrix and dense collagen in the autograft group (Figure 4(l)), negligible staining in the blank control group (Figure 4(k)), which comprised of fibrous and adipose tissue. The BMP2 + ACS group presented a dense composite of proteoglycan matrix and collagen (Figure 4(m)), whereas both Laponite groups the trabeculae were predominantly staining for collagen (Figure 4(n) and (o)) with negligible proteoglycan staining.

The autograft implanted group expressed a high degree of both osteoid (red/orange) and mineralized bone (green) (Figure 4(q)), negligible staining in the control blank group with any new bone formed depicting as osteoid (Figure 4(p)). In the BMP2 + ACS group (Figure 4(r)), the trabeculae expressed a combination of osteoid and mineralized mature bone. New bone in the BMP2 + ACS + LLG group (Figure 4(s)) was comprised of mineralized bone but the new bone in the BMP2 + HLG + ACS group expressed a high amount of osteoid on the trabecular region of the defect site (Figure 4(t)).

**Figure 5.**
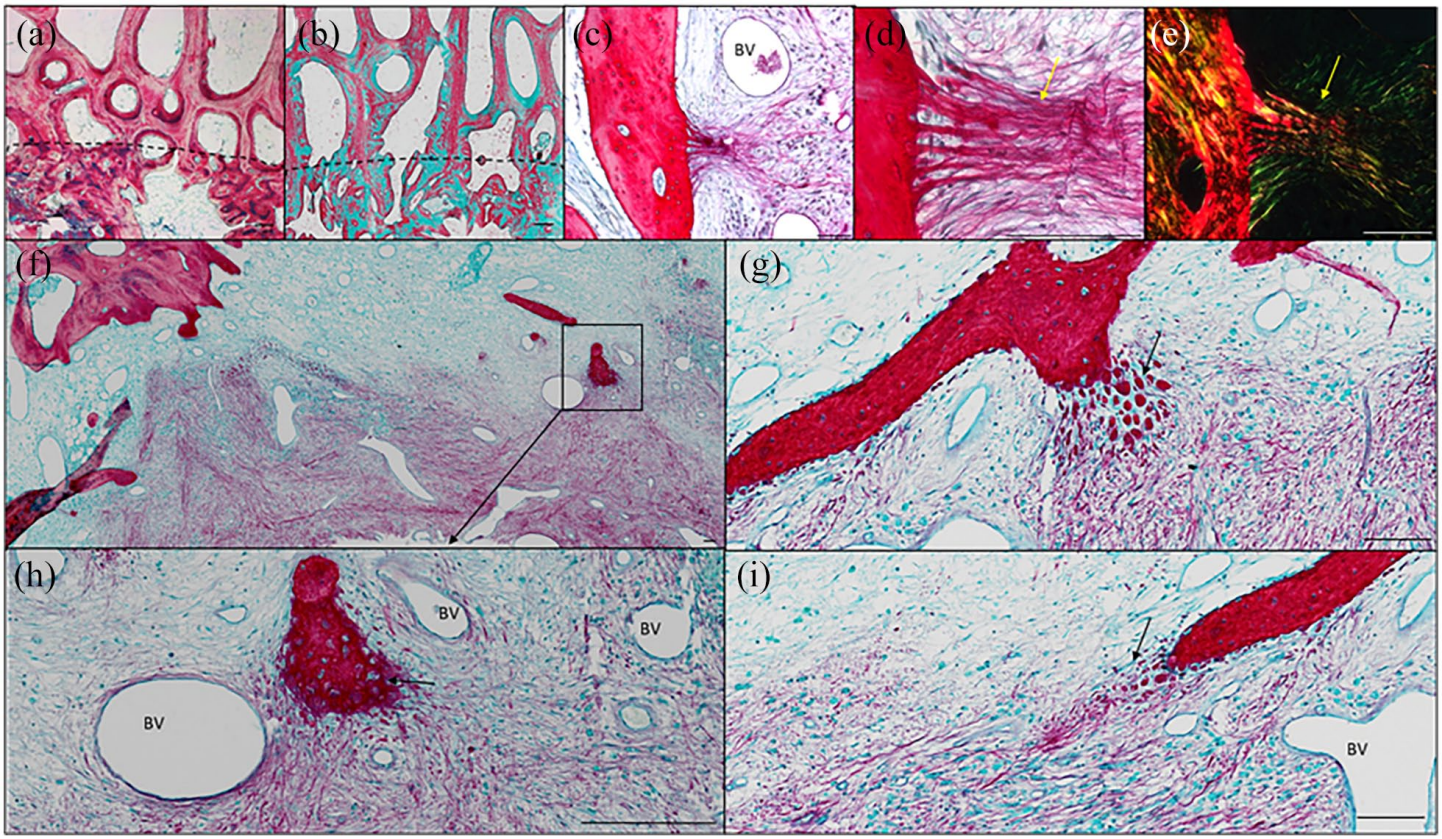
Histological sections of Autograft group stained for Alcian Blue/Sirius red (a) and Goldners trichrome (b) depicting the integration boundary (dotted line) of autograft with native trabecular bone. Organized skeletal fibres (alcian blue/Sirius Red stain) extending from the bone (yellow arrows) into the implant of BMP2 + ACS + LLG (c and d). Polarized light microscopy depicting the organized collagen structures (e). Alcian blue/Sirius red staining of the matrix juxtaposed to the bone trabecular and implanted BMP2 + ACS + LLG in the condyle defect regions (f–i). Black arrows depict packets of new bone formation along with skeletal filaments (g and i), which are of a possible endochondral process (arrow) (h). BV = blood vessel. Scale bar = 100 µm.

In the autograft group there was good integration of the autograft with the native trabecular bone (Figure 5(a) and (b)). However, the autograft was highly disorganised, in contrast to the organised trabecular of the native tissue. On closer inspection of the groups, interesting osteogenic activity in the BMP2 + ACS + LLG group was observed, which was not present in the other groups. We found organized skeletal filaments radiating out of the bone into implanted the matrix possibly indicative of Sharpey’s fibres (Figure 5(c) and (d)). This phenomenon was further characterised with polarised light microscopy revealing an organised collagen fibre band extending from the native bone into the defect (Figure 5(e)). In addition, packets of mineralized nodules and filaments were observed radiating out from the native bone into the implanted matrix (Figure 5(f)–(i)), which appeared to be of endochondral nature with chondrocyte lacunae in situ (Figure 5(h) black arrow). Strikingly, there appeared to be a large number of blood vessel ingrowth developing concomitantly with the tissue sites of neo-bone formation areas. (Figure 5(g)–(i)). Critically, no evidence of toxicity, necrosis or inflammation was observed in any of the groups.

### Microstructural and biomechanical characterisation with digital volume correlation

**Figure 6.**
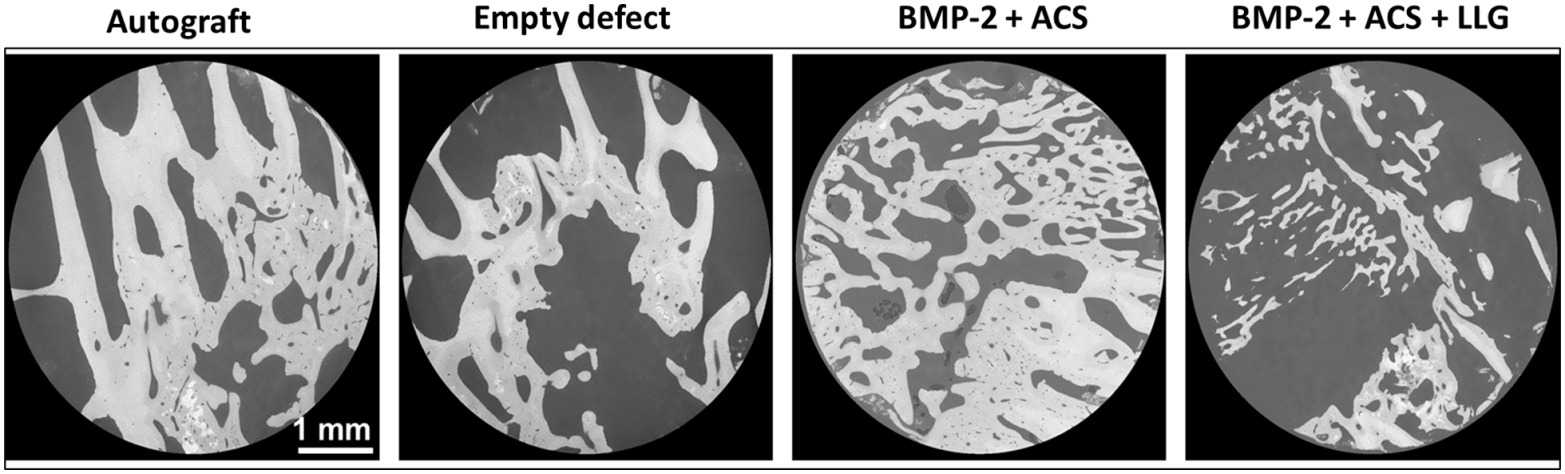
High-resolution XCT cross-sections through the cylindrical cores analysed demonstrating bone formation within the defect regions in each group.

High-resolution XCT images (Figure 6) demonstrated enhanced bone formation with autograft and BMP-2 + ACS compared to blank defects and BMP-2 + ACS + LLG, in agreement with previous morphometric analysis (Figure 3). Large areas of woven bone were identified in autograft and BMP-2 + ACS, whereas larger voids were observed in the blank and BMP-2 + ACS + LLG.

**Figure 7.**
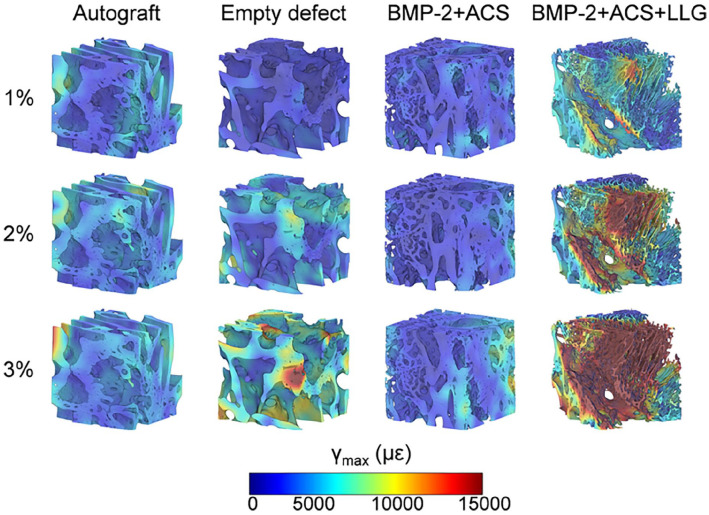
Full-field maximum shear strain distribution (γ_max_) at each compression step for Autograft, Empty defect, BMP-2 + ACS, and BMP-2 + ACS + LLG. A representative volume of interest (3.5 × 3.5 x 3.5 mm^3^), at the centre of each cylinder, is presented to aid enhanced visualization.

The full-field maximum shear strain (γ_max_) for the three loading steps (Figure 7) indicated progressive strain accumulation with the applied compression for all samples. However, the thinner microarchitecture and large voids present within the BMP2 + ACS + LLG produced large and highly strained regions following 3% compression.

The strain distribution in BMP2 + ACS and autograft remained more homogeneous during loading, whereas the blank defect showed discrete areas of localised high strains, particularly, in regions of more immature tissue. The evolution of the internal strain distribution over time (Figure 7), revealed that the major volume of autograft, BMP2 + ACS and blank defects experienced strain levels below 6000 µε and the yielded volume (yield value in shear, γ_max,y_ = 14,500 µε)^29^ did not exceed 1% in any case. Interestingly, significantly higher deformation was observed in the BMP2 + ACS+LLG group, with over 25% of sample volume yielded at 3% compression

## Discussion

The current study has employed an aged ovine model to examine the delivery of a clinically approved growth factor BMP in a range of delivery vehicles including the use of a synthetic nanoclay Laponite to facilitate bone defect repair. An ovine model was developed using only an aged female population of ewes at least 5 years of age, more representative of the clinical demographic in humans at risk of fragility fractures, in marked contrast to the typical use of animals at around 24 months of age.^1,30,31^

The findings demonstrated significant difference in bone volume over empty defect controls only in autograft treatments. Although bone formation did not reach significance compared to autograft alone, our study illustrates the potential of Laponite to deliver active BMP-2 and, importantly the material displayed excellent biocompatibility, with the opportunity for improved efficacy with further optimisation. The enhanced bone formation within the defect observed with autograft compared to the blank control defects validated the experimental model and is consistent with clinical practice. A plethora of variations (defect size, duration, graft used, breed, age of sheep, method analysis) render exact comparisons of this study with other ovine studies challenging. However, our results of bone formation in the blank and control groups are broadly consistent with those reported by Liu et al.^32^

It is important to note that micro-CT assessment of bone volume cannot distinguish between graft material inserted at time of surgery and new bone formation. This is exemplified by U van der Pol and colleagues, who reported a reduction on BV/TV over time in a defect treated with Chronos granules, which, due to their hydroxyapatite nature, are similarly not distinguishable from new bone formation using a standard BV micro-CT endpoint.^21^ Nor does the bone volume endpoint differentiate immature isotropic woven bone, from mature, biomechanically superior anisotropic bone seen in the later stages of healing. Other techniques for assessing bone growth, such as tetracycline double-labelled regions of new bone studied by fluorescence microscopy, can be used to perhaps circumvent these constraints. Further imaging using backscattered electrons (BSE), secondary electrons (SE) and energy dispersive X-ray (EDX) can be utilised to detect small changes in bone microstructure. Critically, in this study greater bone volume from micro-CT analysis was observed to correlate with remodelled bone histologically and biomechanically in the DVC subgroup analysis.

A limitation of this study is the number of samples we were unable to include in the final analysis (Table 1). Early termination of the study in two of the 24 sheep, while unfortunate, is consistent with similar studies.^17,21^ It is important to also note, the selection of aged sheep to facilitate a more representative fragility fracture scenario is likely to have increased the risk of perforation of the cortex or association of the defect with a large void, which was experienced in six cases. Given displacement of graft/test material out of the entire defect (Figure 2) associated in cases of perforation, exclusion from the analysis was deemed justified. Future studies may include the use of a mini-c arm, not available for this study for intraoperative assessment of cortex thickness. We are aware the use of younger sheep could mitigate the risk of cortical perforation, however, a limitation of using younger animals, is the risk of generating and developing a model less representative of bone healing in adult humans. This is likely to be a particular problem if the defect intersects the physis.^33^

The selection of duration for the study is critical, and the ability to select multiple time points limited, necessarily, by the requirement to minimize the number of animals required in keeping with replacement, reduction and refinement in animal research. Our study duration of 10 weeks was informed by other studies^17,21^ and results of positive (autograft BV/TV 47.4%) and negative (blank BV/TV 0.4%) controls support 10 weeks as an appropriate study duration. Nevertheless, it is noteworthy that in a comparable ovine condyle defect U van der Pol et al reported less bone formation with biocomposite test material compared to blank at 2 and 4 months, but greater bone formation with the biocomposite observed at 12 months. The authors themselves commented that had the study duration been 4 months a negative conclusion would have been made.^21^

There is a wealth of literature indicating Bone BMP response and side effects are highly dose dependent, and therefore selection of BMP dose for this study was pivotal. The current work benefitted from previous observations by Liu and Cipitria^32,34^ that indicated that BMP doses around 0.2 and 0.5 mg/cm^3^ were capable of mediating bone formation in an ovine model. The choice of the lower end of this BMP range was based on our recently published murine work, which demonstrated improved BMP efficacy with Laponite^6^ and, in marked contrast, to typical clinical doses 0.5–6 mg/cm^3^ (6–12 mg total dose). This study has demonstrated that InductOs^®^ is capable of mediating bone formation within an ovine defect, at doses lower than those routinely used in humans. This has significant implications for clinical practice, indicating a reduction in doses applied clinically may continue to be efficacious while reducing adverse dose-dependent side effects.

Interestingly and in keeping with our own and other studies,^11^ Laponite was found to be biocompatible with no evidence of inflammation, tissue necrosis or systemic illness associated with use in this large animal study of 10 weeks duration. While Laponite/BMP gels were not found, in this study, to produce significantly greater bone formation within the defect compared to the blank control; micro-CT reconstructions demonstrate bone formation outside the defect, indicating that Laponite was indeed able to deliver active BMP but was displaced from the defect site. Within the defect site itself, histology showed heterogeneous distribution of bone in the Laponite groups. Given the capacity of Laponite to bind and localise BMP2, the distribution of Laponite when combined with other scaffold components is of critical importance. In the current study two alternative constructs were explored both of which may have limited BMP2 distribution throughout the defect site. In the low volume Laponite group (BMP2 + ACS + LLG), where Laponite gel was sandwiched between ACS sponges, BMP2 distribution may have become localised to these low volume regions of Laponite gel. It is notable in this regard that the highly heterogenous distribution of bone in these implants aligned with the restricted distribution of Laponite gel (Supplemental Figure 1). In the high volume Laponite group (BMP2 + HLG + ACS), while BMP2 was distributed through the bulk volume, the absence of BMP2 in the ACS cap placed prior to closure may have limited the periosteal component of bone formation that appeared to make a substantial contribution to bone formation in the BMP2 + ACS control. We also note that the Laponite used in this study was 2.5% dilution by weight, which results in a relatively low gel stiffness. Increasing the stiffness of the Laponite gel can be readily achieved using an increase in Laponite concentration. It is likely that such alterations in Laponite formation could result in superior retention at the target site and greater bone formation and such approaches are currently under evaluation in our group.

The limited DVC analysis preformed confirmed Laponite displacement resulting in enhanced heterogeneous bone formation. A more homogeneous strain distribution was observed for the BMP2 + ACS, in comparison to the Laponite-induced tissue (Figure 7). This suggests load transfer was more efficient in the bone formed via BMP2 + ACS, where strain at the tissue was mainly contained into physiological values (region of 1500 µε^35^). BMP2 + ACS + LLG produced thinner trabeculae and larger regions of void, as previously shown in.^23^ As a result, highly strained regions were observed and developed from the early stages of compression (1%–2%). This finding could represent a potential problem in the clinical setting in two different ways. On one hand, fracture may develop from such strained regions as a result of damage accumulation in case of overloading and progress to failure^36^; thus, jeopardising surgical intervention. On the other hand, the high strain environment in the defect region and the absence of bone-bridging in some areas could lead to inappropriate tissue differentiation, as postulated in the mechano-regulation theory of Prendergast.^22^ Thus, even at larger time points, the mechanical stimuli would not lead to increased ossification and remodelling but formation of fibrous tissue, as observed histologically (Figure 5). However, with the limited numbers analysed with DVC, caution around over interpretation is advocated, as the limited amount of newly formed bone did not allow to extract a larger cohort of specimens of a suitable size for mechanical testing.^37,38^ Yet, the variations in the morphological parameters (i.e. BV/TV) provided a good representation of the differences observed between groups (Figure 3). In addition, this analysis may be combined with computational models^39^ in larger sample sizes and whole defect regions to improve the current understanding between bone regeneration achieved in vivo by the action of different biomaterials and mechanical competence. Nevertheless, importantly, the analysis corroborated the morphometric data obtained on a larger cohort. The findings from the autograft and blank controls validated this femoral ovine condyle defect model. While Laponite/BMP formulation did not mediate greater BV compared to blank, it is likely the Laponite viscosity can be further optimised following the insights afforded from this initial first large animal study.

In summary, the current studies illustrate the potential of an aged ovine bone defect model, more representative of the clinical demographic in humans at risk of fragility fractures, to evaluate delivery and efficacy of bone agents and materials. The aged ovine model demonstrated autograft mediated enhanced bone formation over blank controls, while no significant difference was observed in either Laponite gel-based preparation. While bone formation, did not reach significance using Laponite compared to autograft alone, our study illustrates the potential of Laponite to deliver active BMP-2 and that importantly, the material displayed excellent biocompatibility, with the opportunity for improved efficacy with further optimisation. The enhanced bone formation within the defect observed with autograft compared to the blank control defects validated the experimental model and is consistent with clinical practice. The current studies auger well for the development of bone formation protocols using nanoclay delivery platforms and a clinically proven growth factor in a clinically relevant large animal model, with significant implications therein for translational bone repair.

## Supplemental Material

sj-docx-1-tej-10.1177_20417314221113746 – Supplemental material for Comparison of bone formation mediated by bone morphogenetic protein delivered by nanoclay gels with clinical techniques (autograft and InductOs®) in an ovine bone model Click here for additional data file. Supplemental material, sj-docx-1-tej-10.1177_20417314221113746 for Comparison of bone formation mediated by bone morphogenetic protein delivered by nanoclay gels with clinical techniques (autograft and InductOs®) in an ovine bone model by Cameron Black, David Gibbs, Josephine McEwan, Janos Kanczler, Marta Peña Fernández, Gianluca Tozzi, Jonathan Dawson and Richard Oreffo in Journal of Tissue Engineering
