# Supplementary material for: Comparison of bone formation mediated by bone morphogenetic protein delivered by nanoclay gels with clinical techniques (autograft and InductOs®) in an ovine bone model
Source: J Tissue Eng. 2022 Sep 16;13:20417314221113746. doi: 10.1177/20417314221113746 (PMC9486279; doi:10.1177/20417314221113746)
Supplement: sj-docx-1-tej-10.1177_20417314221113746 – Supplemental material for Comparison of bone formation mediated by bone morphogenetic protein delivered by nanoclay gels with clinical techniques (autograft and InductOs®) in an ovine bone model [file sj-docx-1-tej-10.1177_20417314221113746.docx]

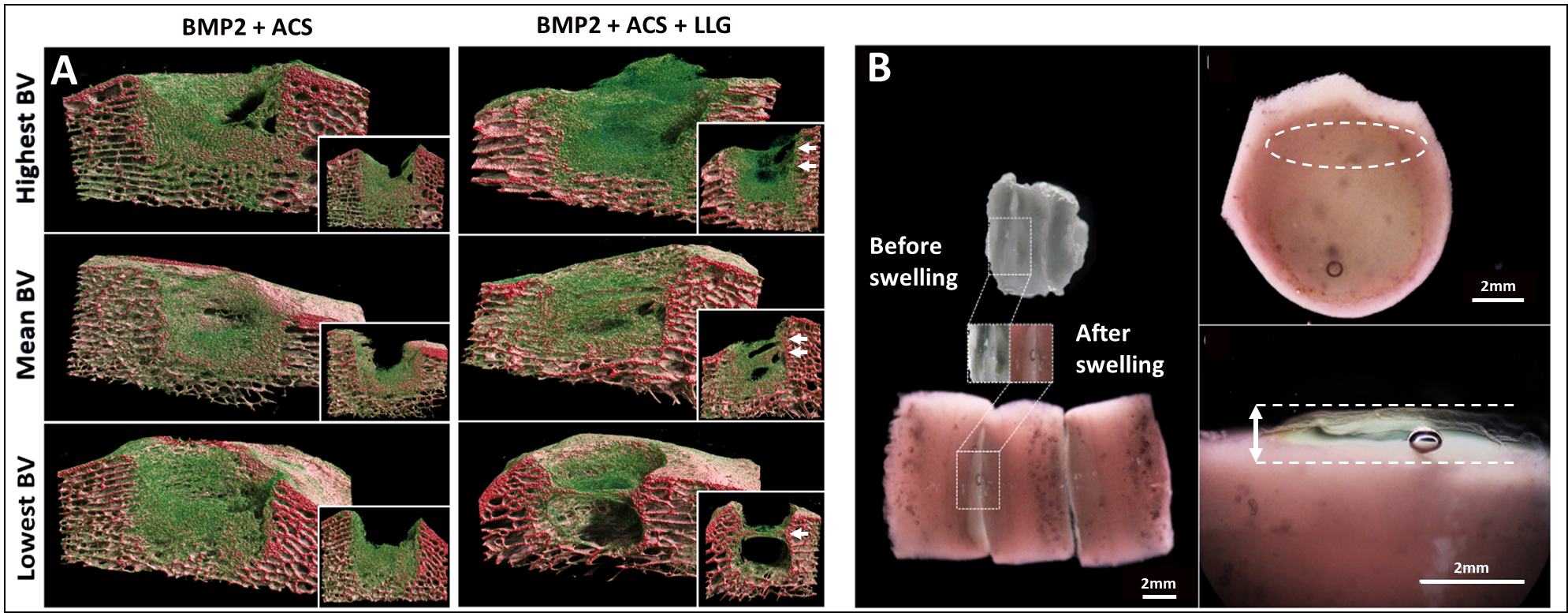


**Supplementary Fig 1: Heterogenous bone and Laponite gel distribution in BMP2 + ACS + LLG implants**. A notable feature of BMP2 + ACS + LLG implants not apparent in BMP2 + ACS alone was the presence of large regions of void intersected with bridging bone (A). These features may reflect the heterogenous distribution of Laponite gel within the defect caused by poor perfusion of swelling ACS by the non-swelling Laponite gel (B).
